# Dysregulated C1q and CD47 in the aging monkey brain: association with myelin damage, microglia reactivity, and cognitive decline

**DOI:** 10.3389/fimmu.2024.1426975

**Published:** 2024-09-27

**Authors:** Sarah A. DeVries, Christina Dimovasili, Maria Medalla, Tara L. Moore, Douglas L. Rosene

**Affiliations:** ^1^ Laboratory for Cognitive Neurobiology, Dept of Anatomy & Neurobiology, Chobanian and Avedisian School of Medicine, Boston University, Boston, MA, United States; ^2^ Laboratory of Neural Circuits and Ultrastructure, Dept of Anatomy & Neurobiology, Chobanian and Avedisian School of Medicine, Boston University, Boston, MA, United States; ^3^ Center for Systems Neuroscience, Boston University, Boston, MA, United States; ^4^ Laboratory of Interventions for Cortical Injury and Cognitive Decline, Dept of Anatomy & Neurobiology, Chobanian and Avedisian School of Medicine, Boston University, Boston, MA, United States

**Keywords:** innate immune system, complement system, neurodegeneration, white matter, microglia, C1q, CD47

## Abstract

Normal aging, though lacking widespread neurodegeneration, is nevertheless characterized by cognitive impairment in learning, memory, and executive function. The aged brain is spared from neuron loss, but white matter is lost and damage to myelin sheaths accumulates. This myelin damage is strongly associated with cognitive impairment. Although the cause of the myelin damage is not known, microglia dysregulation is a likely contributor. Immunologic proteins interact with microglial receptors to modulate microglia-mediated phagocytosis, which mediates myelin damage clearance and turn-over. Two such proteins, “eat me” signal C1q and “don’t eat me” signal CD47, act in opposition with microglia. Both C1q and CD47 have been implicated in Multiple Sclerosis, a demyelinating disease, but whether they play a role in age-related myelin pathology is currently unknown. The present study investigates C1q and CD47 in relation to age-related myelin degeneration using multilabel immunofluorescence, RNAscope, and confocal microscopy in the cingulum bundle of male and female rhesus monkeys across the lifespan. Our findings showed significant age-related elevation in C1q localized to myelin basic protein, and this increase is associated with more severe cognitive impairment. In contrast, CD47 localization to myelin decreased in middle age and oligodendrocyte expression of *CD47* RNA decreased with age. Lastly, microglia reactivity increased with age in association with the changes in C1q and CD47. Together, these results suggest disruption in the balance of “eat me” and “don’t eat me” signals during normal aging, biasing microglia toward increased reactivity and phagocytosis of myelin, resulting in cognitive deficits.

## Introduction

Advancing age is the largest risk factor for the development of many diseases, including neurodegenerative diseases such as Alzheimer’s disease (AD) (1). Beginning in the third decade, decline of several cognitive domains including processing speed, memory, learning, and executive function begins and continues to deteriorate linearly across the lifespan (2–5). This progressive decline manifests as noticeable cognitive deficits with advancing age, and despite the absence of pathology indicative of neurodegeneration or AD, severe cognitive impairment affects 30% of the aged population and interferes with activities of daily living (3). With the population over 65 years increasing, it is important to identify the underlying mechanisms driving age-related cognitive decline so therapeutic interventions targeting them can be developed. In contrast to neurodegenerative diseases like AD or Parkinson’s, neuron loss does not occur with normal aging (6). Nevertheless, neurons in the aging brain exhibit pathological changes that likely interfere with normal connectivity. Namely, breakdown of white matter occurs with age and correlates with cognitive decline (7–9). This myelin damage is evidenced by loss of white matter volume and decreased myelin integrity shown with increasing white matter hyperintensities, particularly in frontal white matter tracts such as the corpus callosum and cingulum bundle (10, 11).

Little is known about the mechanisms underlying myelin degradation with age. However, ultrastructural studies of the rhesus monkey model of normal human aging have shed light on the specific defects aging myelin exhibits. Due to their long lifespans, age-related cognitive decline, and comparable white matter tracts to humans (12–16) our monkey model enables the investigation of how normal aging affects myelin in white matter tracts such as the corpus callosum and cingulum bundle with high translatability to human aging white matter and associated cognitive impairment. Electron microscopy studies in our laboratory show significant myelin degeneration with age in frontal white matter areas including myelin loss or thinning as well as structural changes such as sheath splitting, the presence of less compact myelin, redundant myelin sheaths, and myelin balloons filled with degenerating cytoplasm or fluid (17, 18). Further, loss of myelinated axons have been found in the white matter tracts of aging monkeys (19, 20). An accumulation of these myelin defects with age likely results in axons becoming at least partially demyelinated, causing action potential failures and/or decreased conduction velocity, leading to disrupted cortical communication (21, 22).

Although the cause of myelin pathology is not known, microglia are likely contributors as they play an important role in removing myelin debris that inhibits remyelination (23), a process which becomes dysregulated with age (24). Microglia become overburdened with degenerating myelin, and failure to clear the debris results in accumulation of damaged myelin that blocks remyelination and proper myelin maintenance (23). Moreover, the aging brain is characterized by chronic inflammation (25–27), which is especially elevated in white matter regions (28). This neuroinflammation puts microglia into a cycle of contributing to inflammation while also responding to proinflammatory signaling (29, 30). Chronic neuroinflammation heightens microglia-mediated elimination of cellular components, which may become misdirected with excess pro-inflammatory signaling. The timing and precision of microglia-mediated debris removal is regulated by immunologic proteins that either initiate or inhibit phagocytosis. Two such proteins are the “eat me” classical complement initiator, C1q, and the “don’t eat me” immune-regulatory protein, CD47 (31). Dysregulation in both C1q and CD47 have been implicated in age-related diseases such as Multiple Sclerosis (MS), a chronic demyelinating disease (32, 33), synapse removal (34, 35), and normal aging (36). Our previous study showed that C1q and CD47 expression are dysregulated in aging gray matter, likely contributing to age-related synapse loss (37). Changes in C1q and CD47 in aging white matter may direct microglia towards chronic phagocytosis and inflammation and hinder efficient debris clearance and myelin maintenance. However, studies in white matter have mainly focused on either “eat me” or “don’t eat me” proteins and not both, so the interaction between the two in white matter remains unknown even though both C1q and CD47 bind to myelin (38, 39) and proper balance between the two molecules is critical for phagocytosis (40). Since these signals have not been studied in aging white matter tracts in relation to myelin damage and related cognitive impairment, the present study aimed to assess changes in the balance of C1q and CD47 in the white matter of the aging cingulum bundle in cognitively assessed nonhuman primates. We hypothesized that aging myelin would express less CD47 and more tagging by C1q, which would be robustly increased in the age-related inflammatory environment. Together this would promote phagocytic and inflammatory microglia response, making myelin more vulnerable to phagocytosis. To assess this, RNAscope and multilabel immunofluorescence (IF) were used to analyze age-related changes in the amount of C1q and CD47 as well as their colocalization with myelin and microglia phenotypes in the monkey brain across normal aging.

## Methods

### Subjects

**Figure 1:**
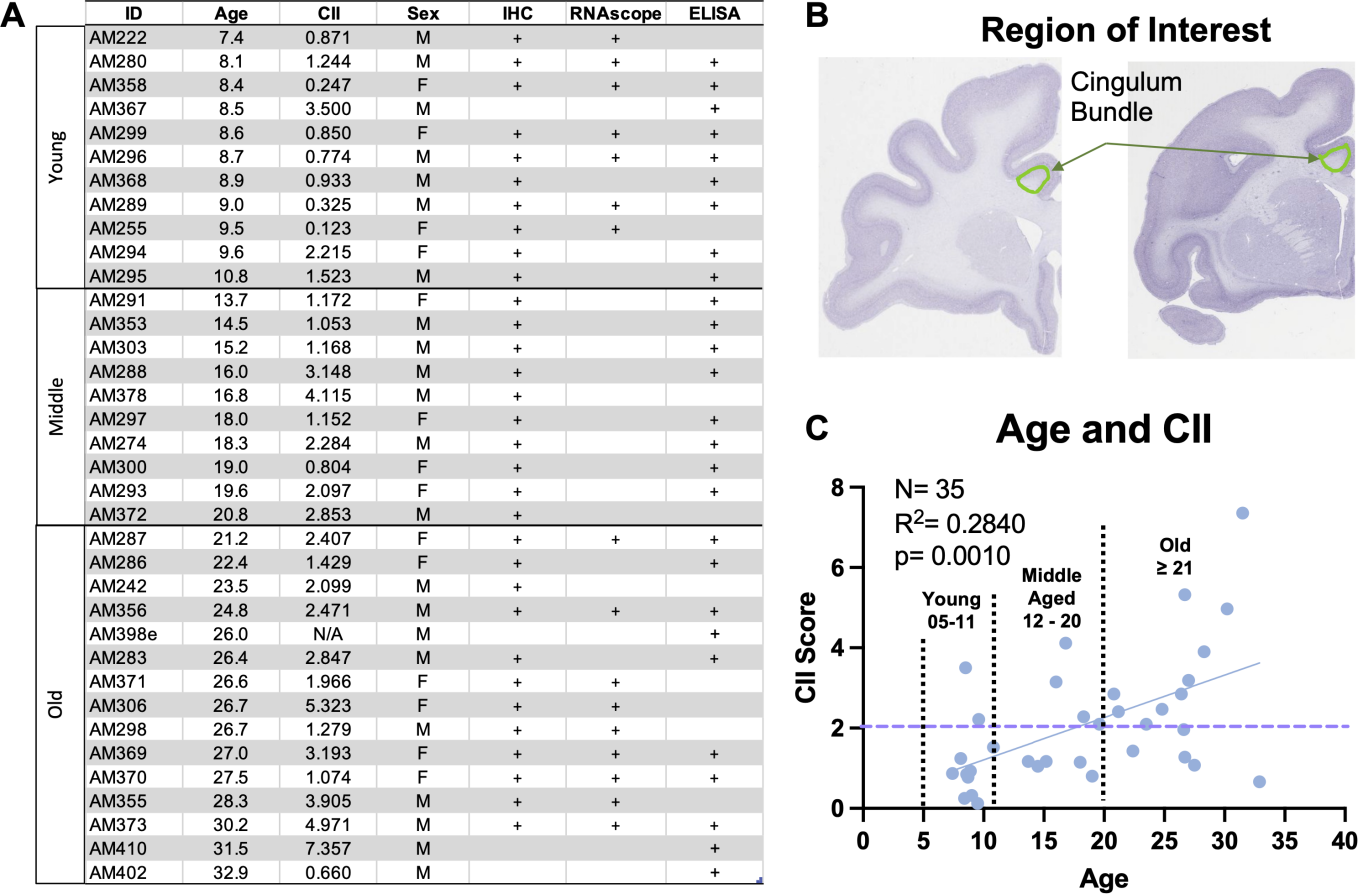
Summary of subject information and experimental parameters. **(A)** Table detailing subject ID, age, sex, and CII score for this study’s cohort of monkeys and defining which samples from each monkey were used for experiments. **(B)** Histological sections showing the cingulum bundle region of interest. **(C)** Graph showing the distribution of cognitive impairment index (CII) score for all 36 animals. Linear regression analysis revealed significant cognitive impairment with age. Stratification of age groups is shown on the graph, where young are aged 5-11, middle age 12-20, and old are >21 years old. Purple dashed line indicates the cut off for cognitive impairment, with scores above 2.0 considered severely impaired and scores below considered cognitively spared.

Brain tissue and CSF samples came from 36 male and female rhesus monkeys ranging from 7-32 years old (**Figures 1A, B**). Tissue from 32 of these animals was used in a previous aging study investigating C1q and CD47 in gray matter (37). All monkeys were behaviorally tested on a battery of tasks to assess learning, memory, and executive function. Testing was done each day prior to once daily feeding of rationed chow, fruit, vegetables, and forage feed. Water was available *ad libitum*. Subjects were maintained in the Animal Science Center on Boston University Medical Campus (BUMC), which is fully accredited by AAALAC and managed by a licensed veterinarian and trained staff. All procedures conformed to the NIH Guide for the Care and Use of Laboratory Animals and were approved by the Boston University Institutional Animal Care and Use Committee (IACUC; protocol number 201800053).

### Cognitive testing

A battery of cognitive tests was administered to all monkeys assessing rule learning, recognition memory, working memory capacity, and executive function. Specific tasks included the delayed non-match to sample (DNMS) acquisition and 120-second delay, the spatial modality of delayed recognition span task (DRST-spatial), and the category set shifting task (CSST). Procedures for these tasks are summarized below but details can be found elsewhere (14, 41–45). Each behavioral task is analyzed individually, but to characterize overall cognitive status of each monkey, a cognitive impairment index (CII) is also calculated by converting scores from DNMS acquisition and 120-second delay and DRST-spatial to z scores relative to a reference group of 29 young adults so that higher scores reflect greater impairments (41). Monkeys with CII scores <2.0 are classified as cognitively spared while those with scores >2.0 are considered cognitively impaired (14). The spread of CII scores for this cohort of monkeys shows significant cognitive impairment with age (**Figure 1C**).

### Delayed non-match to sample

DNMS assesses rule learning and recognition memory. During the acquisition phase, subjects first learn to correctly identify a novel object from a familiar object presented 10 seconds prior. Once correct identification reaches a criterion of 90% correct responses over 100 trials, subjects transition to DNMS-120 second delay. This introduces a 120 second delay between initial sample presentation and choice to assess the recognition of the novel object.

### Delayed recognition span task

DRST-spatial assesses working memory capacity by requiring the subject to identify a new position among an increasing number of spatial locations. For this task, subjects are presented with identical objects for all trials. With each trial, one additional object is added to a novel location among previously presented locations. Subjects must remember the location of all previously presented items and correctly identify the object at the novel location. Scoring is based on the number of locations chosen correctly prior to an error.

### Category set shifting task

This task is an adaptation of the human Wisconsin Card Sorting task and measures aspects of executive function including cognitive set shifting, abstraction, and perseveration. For this task, subjects learn to respond to a certain stimulus property (color or shape), and once the rule is learned, they must learn to shift to a new stimulus category without warning to receive reward (43, 44). CSST provides numerous outcome measures, but we chose to analyze the total number of perseverative errors and broken sets out of total errors based on previous research in our laboratory showing these measures are sensitive to age-related changes (43, 44, 46).

### Cerebrospinal fluid collection

Immediately prior to euthanasia, cerebrospinal fluid (CSF) was extracted from the cisterna magna with the monkey sedated with 10mg/kg Ketamine. The sample was frozen immediately at -80°C for long term storage. 

### Euthanasia and brain harvesting

After completing cognitive assessment, subjects were euthanized and perfused with a two-step protocol that allows both fresh and fixed brain tissue to be harvested, as described (47, 48). Monkeys were deeply anesthetized with Sodium Pentobarbital (25 mg/kg to effect) and euthanized by exsanguination during transcardial perfusion fixation of the brain. Once the animal is deeply anesthetized, the chest cavity is opened, and 4°C Krebs buffer is transcardially perfused via the ascending aorta to flush vasculature and rapidly cool the brain during which the left hemisphere is removed and cut into 2mm thick slabs in a brain matrix and promptly frozen at -80°C to limit proteolysis. Once the left hemisphere is removed, the perfusate is switched to 4% paraformaldehyde (PF) at 37°C for 10 minutes to ensure full fixation. The fixed hemisphere is blocked in situ in the coronal plane, postfixed overnight in 4% PF at 4°C, and cryoprotected in 10% and then 20% glycerol with 2% DMSO and 0.1M buffer (47) before being flash frozen in isopentane at -75°C and stored at -80ºC until it is cut into 10 interrupted series of 30µm sections. These sections are collected into a buffer with 15% glycerol and stored at -80°C until removed for processing, which preserves integrity for histochemical processing (48). One series is stored with the addition of 1% PF to ensure optimal RNA preservation for RNAscope studies.

### Immunofluorescence

To assess the amount of myelin, C1q, and CD47 present in the cingulum bundle, as well as colocalization of both C1q and CD47 to myelin, immunofluorescence (IF) was performed. Antibodies against C1q and CD47 were used in addition to myelin basic protein (MBP) as a myelin marker. MBP was chosen as it accounts for 30% of myelin composition and mediates tight lamination between myelin lamellae (49) Additionally, IF was used to assess the morphology of microglia using the pan-microglia marker Iba1 and microglia expression of phagocytic protein Galectin-3 (Gal-3) and complement protein C1q. Gal-3 is required for phagocytosis in microglia, is upregulated during aging and neurodegeneration, and may be involved in myelin debris clearance (50). Together, these experiments aimed to reveal changes in C1q and CD47 in relation to myelin loss and increased microglia reactivity.

For IF, fixed tissue from 32 animals was removed from -80°C storage, thawed, and batch processed. For each animal, 3 tissue slices containing the anterior cingulum bundle spaced 2400µm apart were used (**Figure 1B**). To begin, sections were washed in 0.05M TBS (pH 7.60) for 3x5 min to remove storage buffer. This was followed by antigen retrieval treatment with 10mM sodium citrate buffer (pH 6.0) in a microwave tissue processor (PELCO Biowave, Ted Pella, Inc. Redding, CA) for 5 min at 550W and 50°C to break cross links that may have formed during fixation. After tissue returned to room temperature, sections were blocked in 10% normal goat serum (NGS; Sigma-Aldrich) and 0.4% Triton-X (Tx) in TBS for 1 hr at room temperature. Tissue was then incubated in 2% NGS and 0.2% Tx in TBS along with appropriate mixtures of the following primary antibodies: chicken MBP (1:400; Millipore Sigma, cat# AB9348), mouse C1q (1:400; Abcam, cat# ab71940), rabbit CD47 (1:500; Abcam, cat# ab218810), rabbit Iba1 (1:500; Wako cat# 019-19741), and rat Gal-3 (1:400; ThermoFisher, cat# 14-5301-82) for 72 hrs at 4°C. To enhance antibody penetration, tissue in primary antibody solution was treated in the microwave tissue processor at 150W, 30°C for 2x5 min and allowed to return to room temperature for 1 hr prior to placing in 4°C. To ensure specificity, additional sections were run as needed omitting primary antibodies. On day two, tissue was rinsed with 0.05M TBS and incubated in 2% NGS and 0.2% Tx solution with AlexaFluor 488 goat anti-chicken IgY (1:500; Thermo Fisher Scientific, MA), AlexaFluor 568 goat anti-mouse IgG2b (1:500), AlexaFluor 647 goat anti-rabbit IgG (1:500), AlexaFluor 488 goat anti-rabbit IgG (1:500), and AlexaFluor 647 goat anti-rat IgG (1:500) for 2 hrs at room temperature. Sections were mounted, coverslipped with antifading polyvinyl alcohol DABCO (Sigma-Aldrich, #10981) mounting media, and stored at 4°C until removed for imaging.

Images were acquired on a Zeiss LSM 710 NLO confocal microscope with a 40X oil-immersion objective lens. A 0.5mm^2^ tilescan was captured in the center of the cingulum bundle and a grid was overlaid onto the tilescan using ImageJ and images were systematically randomized for analysis. Selected images were thresholded to the same numbers for each channel, then the global % area in the cingulum bundle was measured on ImageJ and colocalization was analyzed using the Colocalization Threshold plugin on ImageJ. One animal was excluded from all analyses for poor antibody penetration across all channels. The density of Iba1+ microglia displaying ramified and hypertrophic morphologies were marked in Neurolucida software (MBF Biosciences) and cells were further marked as C1q+, Gal3+, C1q+Gal3+, or C1q-Gal3-. The proportion of each classification was calculated.

### RNAscope

A subset of 16 male and female subjects were stratified according to age as young (<11 years; n=7) or old (>20 years; n=9) and cognitive characterization regardless of age as spared (<2.0) or cognitively impaired (>2.0) as shown in **Figure 1A** for RNAscope analysis. RNAscope hybridization protocols were carried out using the RNAscope Multiplex Fluorescent Manual Assay kit from Advanced Cell Diagnostics (ACD) according to the manufacturer’s instructions. Using one section per subject from the series saved in 15% glycerol with 1% PF, the cingulum bundle was dissected out and the sections were treated with H_2_O_2_ for 10 min at room temperature, then mounted on SuperFrost Plus microscope slides (Fisher Scientific, MA). After tissue dried and adhered to the slide, target antigen retrieval was performed using Target Retrieval reagent (ACD, CA) at 95°C for 5 min in addition to a 30 min incubation in Protease Plus (ACD, CA) at 40°C in the HybEZ II oven. Tissue was incubated in primary probes *C1qA* (ref# 1200891-C1; ACD, CA), *CD47* (ref# 1200901-C2; ACD, CA), and *Olig2* (ref# 1203071-C1; ACD, CA) for 2 hours at 40°C and stored in 5x SCC hybridization buffer at room temperature overnight. The following day, tissue was incubated in three probe amplification steps (AMP 1-3) and fluorophores Opal 690 (1:300 in TSA buffer; Akoya Biosciences, MA) and Opal 570 (1:750; Akoya Biosciences, MA) were conjugated at 40°C. Two additional slices of tissue were incubated with appropriate ACD probe mixes for a positive control (custom cocktail of #521081, 461341, 457711 housekeeping probes) and a negative control (mix # 320871).

Immediately after RNAscope, tissue was processed for IF with 100% Superblock (Thermo Fisher Scientific, MA) at 40°C for 1 hr, and then incubated in TBS with 0.5% Superblock and 0.3% Tx along with rabbit Iba1 primary antibody (1:250; Wako, cat# 019-19741) for 1 hr at 40°C. Tissue was treated with AlexaFluor 488 donkey anti-rabbit fluorescent secondary for 1 hr at 40°C, followed by treatment with DAPI for 30 sec. Finally, slides were coverslipped with Prolong Gold Mounting Medium (Thermo Fisher Scientific, MA).

Images were acquired in a tilescan on a Zeiss LSM 710 NLO confocal microscope using a 40X oil immersion objective lens. Images were analyzed manually by first identifying 130-140 Iba1+ or Olig2+ cells and then counting the *C1qA* or *CD47* RNA puncta located within the DAPI-labelled soma.

### Enzyme-linked immunosorbent assay

To measure MBP in CSF samples (**Figure 1A**), an ELISA was run across age groups. Previous research has shown increased levels of MBP in the CSF in disease states as an indication of increased damaged myelin in the brain which gets removed via clearance into CSF (51, 52). The ELISA was performed according to manufacturer’s instructions for MBP (ANSH Labs, cat# AL108). All CSF samples were thawed from -80°C while on ice and diluted to a concentration of 1:4 with ultrapure water. All reagents, calibrators, and controls were thawed, and the assay was performed at room temperature. All MBP calibrators and controls were reconstituted with 1mL of deionized water each, and all calibrators, controls, and CSF samples were plated in duplicate. Samples were incubated for 1 hr on an orbital microplate shaker at 600rpm. Solution was removed and each well was washed 5 times with wash solution. Following this, the MBP Antibody-Biotin Conjugate Solution was incubated for 1 hr while agitated at 600rpm, after which solution was removed and washed as above. Then Streptavidin Conjugate was added and incubated for 30 min shaking at 600rpm, wells were washed, and TMB chromogen was incubated for 10 min while covered. Finally, stop solution was added, and the absorbance of the solution in the wells was read on a microplate reader (BioRad, Berkeley, California, USA) at a 450nm wavelength.

### Statistics

All analyses were performed with the experimenter blinded to subject identity. Data were analyzed using GraphPad Prism (Version 10.1.1) with an *α* ≤ 0.05. IF data were analyzed by linear regression with age and cognitive impairment (CII score, CSST total broken sets, and CSST perseverative errors/total shift errors) as independent variables and % area of MBP, C1q, CD47, colocalization, and microglia phenotypes as dependent variables. Further analysis between age groups and cognitive impairment classification were analyzed with one-way analysis of variance (ANOVA) followed with Tukey’s *post hoc* test to control for multiple comparisons. Sex and sex by age interaction were analyzed with a two-way ANOVA and Tukey’s *post hoc* test. RNAscope data were analyzed between age groups and cognitive status. Data are shown as box plots and group differences were evaluated with a two-tailed independent samples t-test. Relative concentration of MBP in the CSF was analyzed using a one-way ANOVA with Tukey’s *post hoc* test comparing young, middle, and old age groups.

## Results

### Myelin disruption associated with C1q occurs with age and cognitive impairment

**Figure 2:**
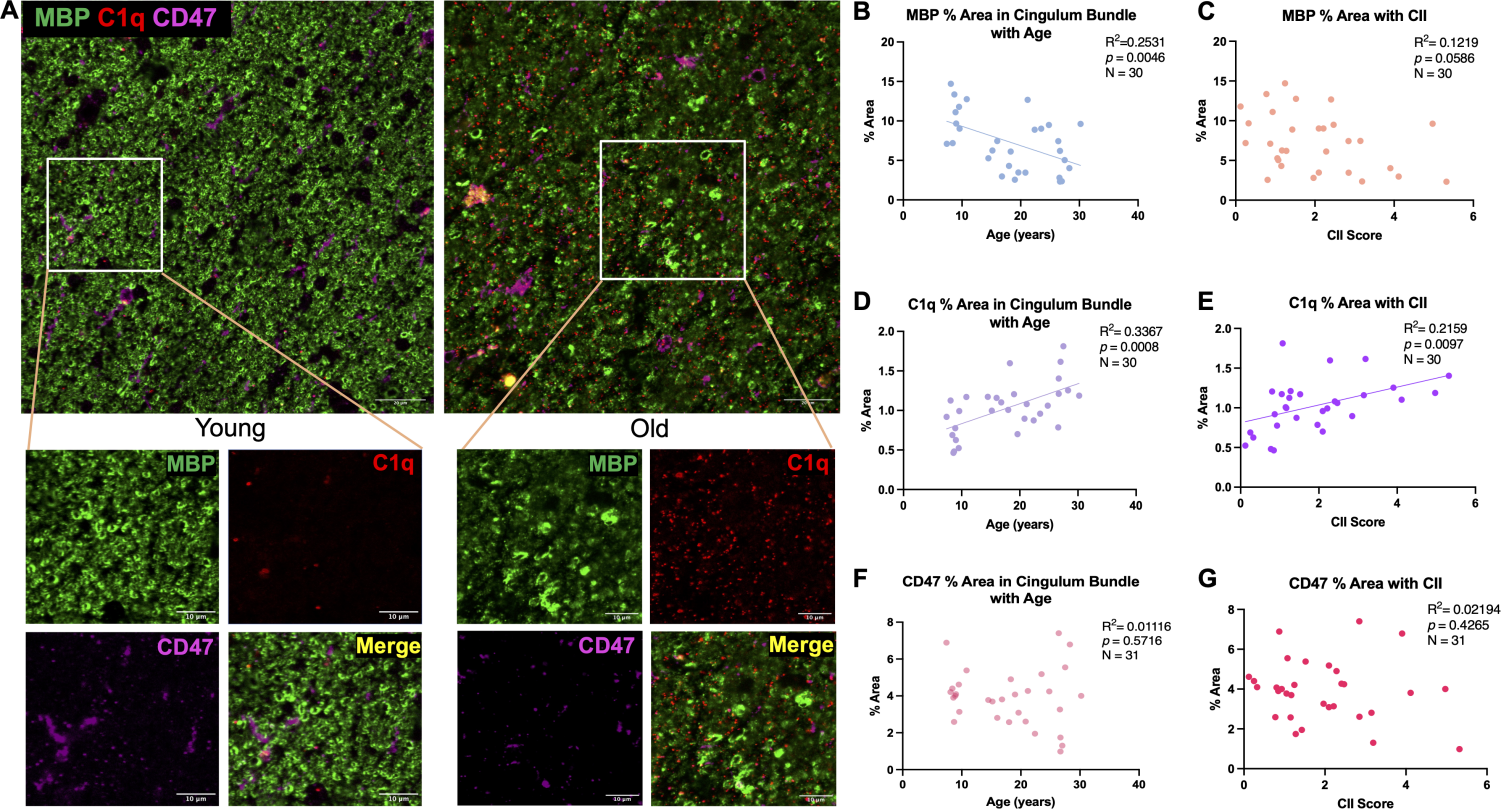
Changes in MBP, C1q, and CD47 in the cingulum bundle with age and CII score. **(A)** Representative immunofluorescent images for MBP, C1q, and CD47 in a young and old animal. **(B)** Significant decrease in % area of MBP was found with age that also correlated with **(C)** cognitive impairment. C1q was significantly increased with **(D)** age and **(E)** CII score but % area of CD47 remained stable with both **(F)** age and **(G)** higher CII score. Scale bars represent 20µm in zoomed out images and 10µm for close-up images.

Myelin damage and loss is well documented in frontal white matter areas during aging (17, 18, 53, 54). Here, MBP, a major component of myelin composition (55), was used to identify myelin together with antibodies against C1q and CD47 in the cingulum bundle, as shown in **Figure 2A**. Two-way ANOVA revealed no sex differences in % area of MBP [F(1,26)= .1409, *p*=0.7104] (data not shown), so data from both sexes was pooled and analyzed together across age in all subsequent analyses. Subject AM299 and AM283 were identified as outliers using the ROUT test on GraphPad prism and were excluded from MBP and C1q analyses, respectively. Results show a significant linear decrease in myelin marked by MBP with age [F(1,28)= 9.488, R^2^ = 0.2531, *p*=0.0046] (**Figure 2B**). The decreases in MBP levels were also associated with increasing cognitive impairment index (CII) score but only approached significance [F(1,28)= 3.888, R^2^ = 0.1219, *p*=0.0586] (**Figure 2C**). Coincident with this, the levels of C1q increased with age [F(1,28)= 14.21, R^2^ = 0.3367, *p*=0.0008] (**Figure 2D**) that was associated with higher CII score [F(1,28)= 7.711, R^2^ = 0.2159, *p*=0.0097] (**Figure 2E**). The % area of MBP and C1q did not correlate with CSST total broken sets or with perseverative errors/total errors (data not shown). Finally, the % area of CD47 did not change with age [F(1,29)= 0.3274, R^2^ = 0.01116, *p*=0.5716] (**Figure 2F**) nor was it related to CII [F(1,29)= 0.6505, R^2^ = 0.02194, *p*=0.4265] (**Figure 2G**). These results confirm previous findings that the myelin is lost in the cingulum bundle with age in addition to structural defects observed (18, 19) and suggest that the increase in C1q may indicate an inflammatory environment priming microglia for phagocytosis.

### Colocalization of C1q with MBP in the cingulum bundle

**Figure 3:**
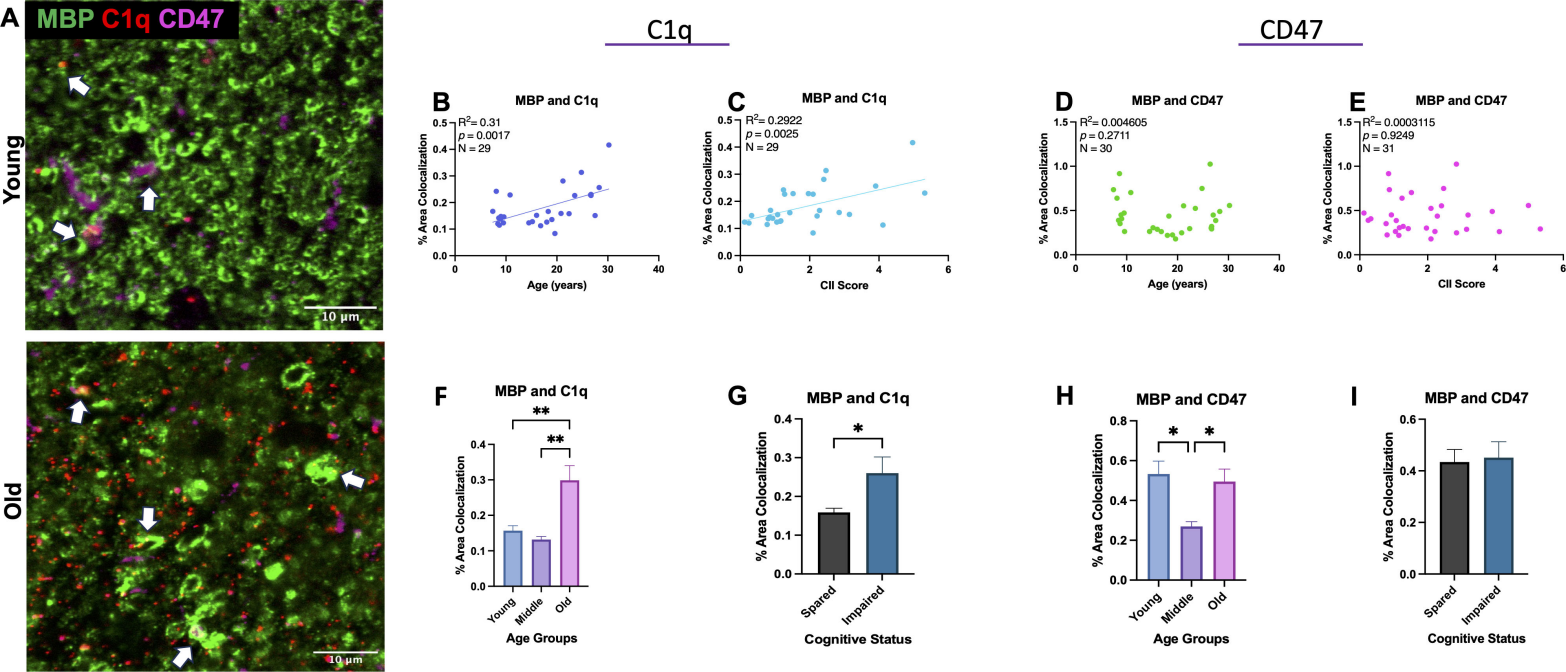
Colocalization of C1q and CD47 with MBP during aging and related cognitive impairment. **(A)** Representative immunofluorescent images of C1q, CD47, and MBP. Colocalization is marked with white arrows. **(B)** C1q and MBP colocalization increased with age and **(C)** CII score but **(D, E)** CD47 colocalized with MBP did not change linearly with age or CII score. **(F, G)** Analysis into age and cognition group differences revealed increased C1q-MBP colocalization in old age compared to young and middle age as well as in cognitively impaired compared to spared. **(H)** Analysis into age groups revealed significant decrease in CD47 localized with MBP in middle age compared to young and old age. **(I)** CD47-MBP colocalization did not correlate with cognitive impairment. Young <10, Old >21; cognitively spared <2.0, cognitively impaired >2.0.

To determine if age-related changes in C1q and CD47 targeted white matter specifically, colocalization of C1q and CD47 with MBP as a marker of myelin was analyzed, as shown in **Figure 3A**. Indeed, results from linear regression analysis revealed a significant increase in C1q-MBP colocalization with age [F(1,27)= 12.13, R^2^ = 0.3100, *p*=0.0017] (**Figure 3B**) that also correlated with CII score [F(1,27)= 11.15, R^2^ = 0.2922, *p*=0.0025](**Figure 3C**). In contrast to C1q, there was no linear correlation between CD47-MBP colocalization and age [F(1,29)= 0.1342, R^2^ = 0.0046, *p*=0.7168] or CII score [F(1,29)=0.00903, R^2 ^ = 0.000311, *p*=0.9249] (**Figures 3D, E**).

To further analyze when these changes occur, we grouped monkeys according to young (<11), middle aged (12-20), and old (21-32) and compared across these age groups. One-way ANOVA with multiple comparison Tukey’s *post-hoc* analyses revealed a significant increase in C1q-MBP colocalization in old monkeys relative to both young (Main effect *p*=0.0005; Tukey’s *post-hoc p*=0.0040) and middle-aged (*post-hoc p*=0.0011). We then assessed these variables relative to cognitive impairment by comparing cognitively spared and cognitively impaired groups across all animals regardless of age. One-way ANOVA revealed higher C1q-MBP colocalization in cognitively impaired animals (CII>2.0) (*p*<0.0001; **Figures 3F, G**). Animals AM283 and AM369 were identified as an outlier using the ROUT test on GraphPad prism and were excluded from C1q-MBP analyses. For CD47-MBP, when discrete age groups were compared with one-way ANOVA, a significant decrease was found in middle-age compared to young (*p*= 0.0111) and old (*p*=0.0245; **Figure 3H**). One-way ANOVA comparison between cognitive classification revealed there was no differences between cognitively spared and impaired animals (*p*=0.5927; **Figure 3I**). Neither % area of C1q-MBP nor % area of CD47-MBP was correlated with CSST total broken sets or perseverative errors (data not shown). Results suggest significant increase in myelin being tagged for phagocytosis by C1q with age, while CD47 expression specifically decreases in middle age, perhaps in response to myelin damage to facilitate repair and remyelination by accelerating myelin debris clearance.

### Microglia exhibit a phagocytic and inflammatory phenotype with age

**Figure 4:**
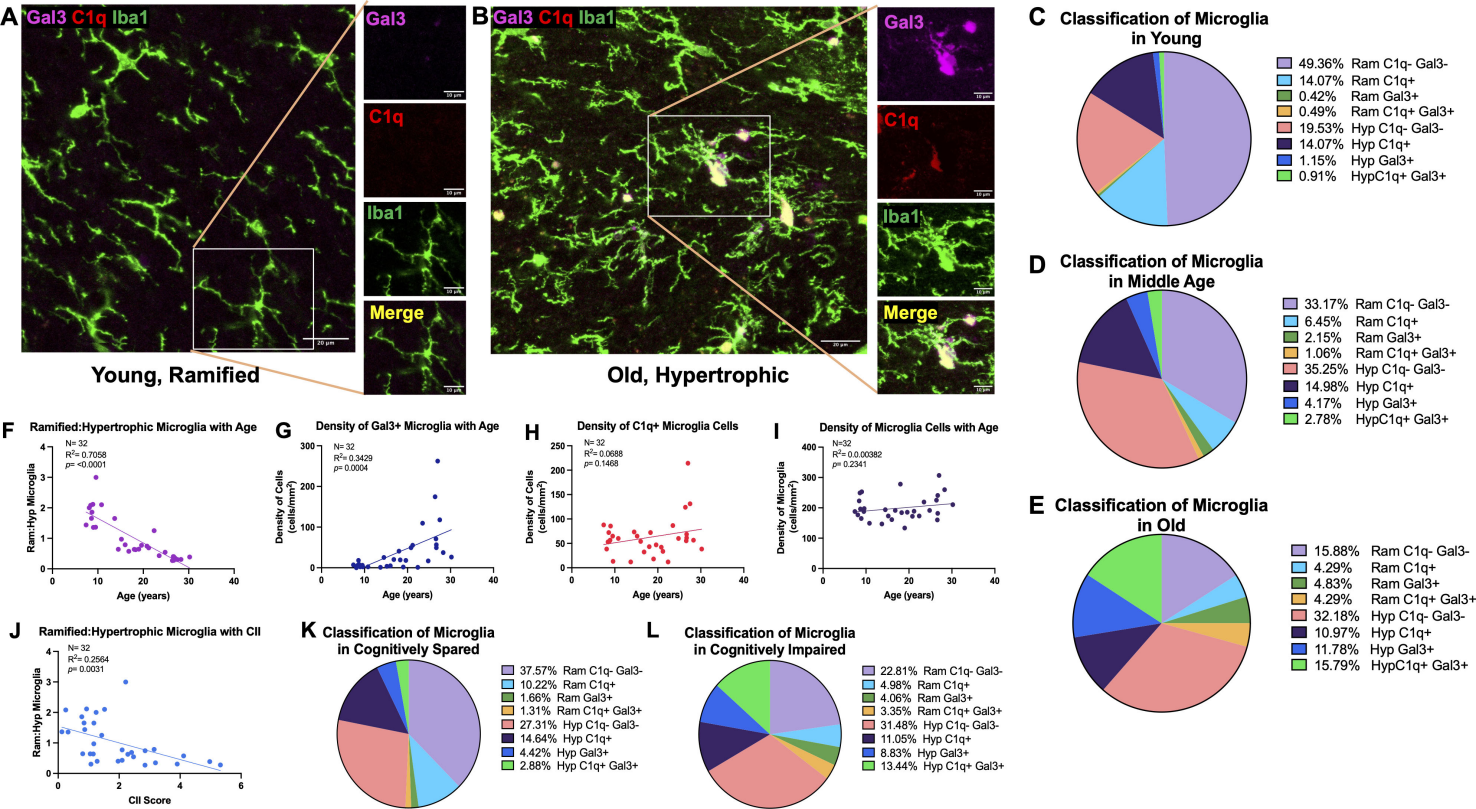
Increased proportion and density of phagocytic and inflammatory microglia with age and cognitive impairment. **(A)** Representative image of Iba1+ microglia in a young animal largely expressing ramified morphology without C1q or Gal-3, as shown on zoomed in image, in contrast to **(B)** old animals with more Iba1+ cells exhibiting hypertrophic morphology with C1q and Gal-3 expression. The proportion of microglia cells classified according to ramified or hypertrophic morphology and expression of C1q and Gal-3 are displayed in **(C)** young **(D)** middle aged **(E)** and old animals. The proportion of ramified: hypertrophic microglia significantly decreased with **(F)** age **(J)** and CII score, indicating an increase in hypertrophic microglia. **(G)** Density of Gal3+ cells increased with age, **(H)** while there were no significant age-related changes in the density of C1q+ cells. **(I)** There was no change in density of total microglia. Proportion of microglia morphology and C1q/Gal3 expression in **(K)** cognitively spared animals and in **(L)** cognitively impaired are also shown. Scale bars represent 20µm in zoomed out images and 10µm for close-up images.

To investigate whether microglia in the cingulum bundle exhibit phenotypic changes in response to changes in C1q and CD47, IF was used with Iba1 to visualize morphology together with expression of the phagocytic marker Gal-3 and inflammatory marker C1q. Microglia were classified according to Karperien’s (56) morphological categories where ramified microglia are considered homeostatic/surveillant and hypertrophic microglia are phagocytic. We therefore used multilabel IF to classify microglia based on Gal-3 and C1q expression and by morphology as follows: Gal3+ramified, Gal3+hypertrophic, C1q+ramified, C1q+hypertrophic, C1q+Gal3+ramified, C1q+Gal3+hypertrophic, C1q-Gal3-ramified, and C1q-Gal3-hypertrophic. Examples of homeostatic and hypertrophic microglia along with Gal3+ and C1q+ staining are shown in **Figures 4A, B** and proportions of phenotypes with age are shown in **Figures 4C–E**. In young monkeys about 50% of the microglia are ramified and Gal3- and C1q-. Interestingly, compared to young, middle aged monkeys had a 4-fold increase and old monkeys a 10-fold increase in the proportion of Gal3+ hypertrophic microglia, indicating increased phagocytosis. This was not the case for C1q+ microglia, which were found in somewhat equivalent proportions across all ages: 29% in young, 25% in middle-aged, and 33% in old.

With age, we found a significant decrease in the ratio of ramified to hypertrophic microglia [F(1,30)= 71.96; R^2^ = 0.7058, *p*<0.0001] (**Figure 4F**), indicating increasing numbers of microglia exhibiting a hypertrophic morphology. Furthermore, results showed a significant increase in the density of Gal3+ cells [F(1,30)= 15.66, R^2^ = 0.3429, *p*=0.0004] but no significant change in density of C1q+ cells [F(1,30)= 2.218, R^2^ = 0.06886, *p*=0.1468] with age (**Figures 4G, H**). Interestingly, both ramified Gal3+ (*p*<0.0001) and hypertrophic Gal3+ (*p*<0.0001) microglia increased with age, but C1q+ ramified decreased with age (*p*<0.0001) and hypertrophic C1q+ microglia did not change with age (*p*=0.7369). Furthermore, the density of C1q+Gal3+ hypertrophic (*p*=0.0062) and hypertrophic C1q-Gal3- microglia (*p*= 0.0023) increased with age. Importantly, there were no changes in the density of total Iba1+ microglia (**Figure 4I**). The decreased ratio of ramified:hypertrophic microglia correlated with higher CII score [F(1,30)= 10.34; R^2^ = 0.2564, *p*=0.0031] (**Figure 4J**) and the increased density of Gal3+ cells approached significant correlation with CII score [F(1,30)=3.263, R^2^ = 0.09810, *p*=0.0809] but density of C1q+ cells did not correlate with cognitive impairment [F(1,30)= 0.1048, R^2^ = 0.003482, *p*=0.7484] (data not shown). The changes in distribution of morphology and protein expression with CII are presented in **Figures 4K, L**. In sum, microglia change from a majority showing ramified morphology that were C1q- and Gal-3- in young age (**Figure 4C**) to more hypertrophic morphology with increased Gal-3 protein expression with old age (**Figure 4E**). Interestingly, middle aged subjects show intermediary profiles suggesting the transition of microglia into more phagocytic and inflammatory phenotypes (**Figure 4D**). Taken together, increased shift to phagocytic and inflammatory microglia phenotypes in the cingulum bundle with age are associated with increased C1q expression on myelin and with more severe cognitive impairment.

### Increased microglial C1qA mRNA expression and decreased oligodendrocyte CD47 mRNA expression in the cingulum bundle with age

**Figure 5:**
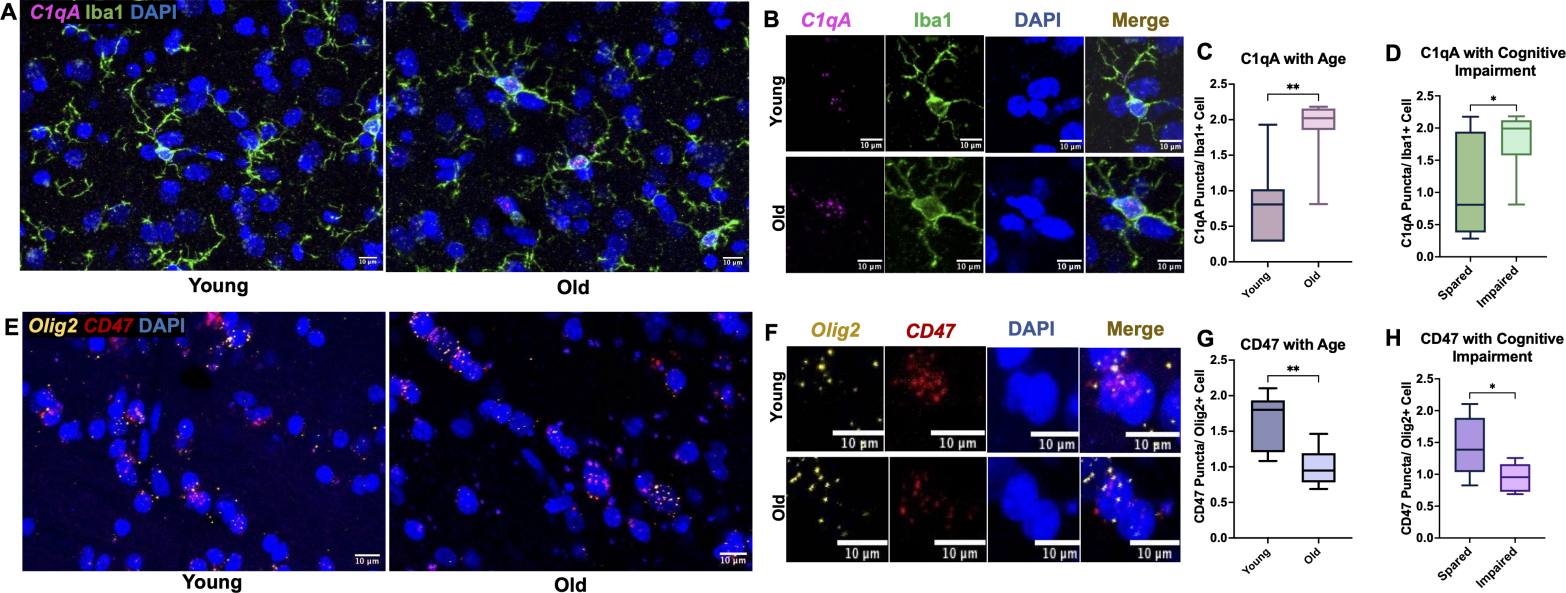
*C1qA* RNA expression increases in microglia cells while *CD47* RNA expression decreases in oligodendrocytes. **(A)** Representative RNAscope images of *C1qA* and DAPI with IF for Iba1 captured in young vs old animals with **(B)** zoomed examples show *C1qA* RNA puncta within single microglia cells. Results show significant increase in *C1qA* RNA expression with **(C)** age and **(D)** with cognitive impairment. **(E)** Example RNAscope images of *Olig2* and *CD47* probes with DAPI in the cingulum bundle of young and old animals. **(F)** Zoomed in images of *Olig2*+ oligodendrocytes expressing *CD47* puncta within DAPI labeled nuclei. **(G)** Decreased *CD47* RNA expression in oligodendrocytes was found in old animals and **(H)** in cognitively impaired animals. *p<0.05; **p<0.01; N=16.

While there was an age-related increase in C1q protein within the cingulum bundle neuropil, we did not observe age-related differences in microglia expression of C1q protein. Indeed, while C1q+ microglia are associated with immune activation in disease models, there is evidence of diverse interactions between C1q+ production, the downstream receptors, and feedback signaling that can promote both pro- and anti-inflammatory microenvironments (57). Thus, we wanted to assess a part of this dynamic process, by looking at RNA expression of genes for C1q in the cingulum bundle. RNAscope was used with a probe for *C1qA*, a subunit ubiquitously found in all C1q molecules (58, 59). RNA expression was analyzed with age and cognitive impairment groups defined as young (<11) or old (>20) and cognitively spared (<2.0) or impaired (>2.0). *C1qA* mRNA puncta were counted inside of immunofluorescent labeled Iba1+ cells, as shown in **Figures 5A, B**. Results showed a significant increase in microglial *C1qA* in old (125.5% increase, *p*= 0.0014; **Figure 5C**) and in cognitively impaired subjects (74.5% increase *p*= 0.038; **Figure 5D**). These results suggest that microglial production of *C1qA* is increased in the aging cingulum bundle.

Given that we see age-related differences in MBP interactions with immune proteins, we then wanted to assess changes in the expression of CD47 RNA within oligodendrocytes, which form and maintain myelin. We hypothesized that oligodendrocytes are less protected by CD47 with age and may be targeted for elimination, leading to disrupted myelination. Another possibility is that oligodendrocytes with less CD47 would produce myelin with less CD47, leaving it more vulnerable to phagocytosis. Here, *CD47* puncta were counted in *Olig2+* cells as shown in **Figures 5E, F**. Results showed a significant decrease in *CD47* in the old age group (38.7% decrease, *p*= 0.0019; **Figure 5G**) and with cognitive impairment (34.3% decrease, *p*= 0.027; **Figure 5H**). These results suggest that oligodendrocytes are less efficient at producing *CD47* with age.

### Elevated levels of MBP in the CSF with age

**Figure 6:**
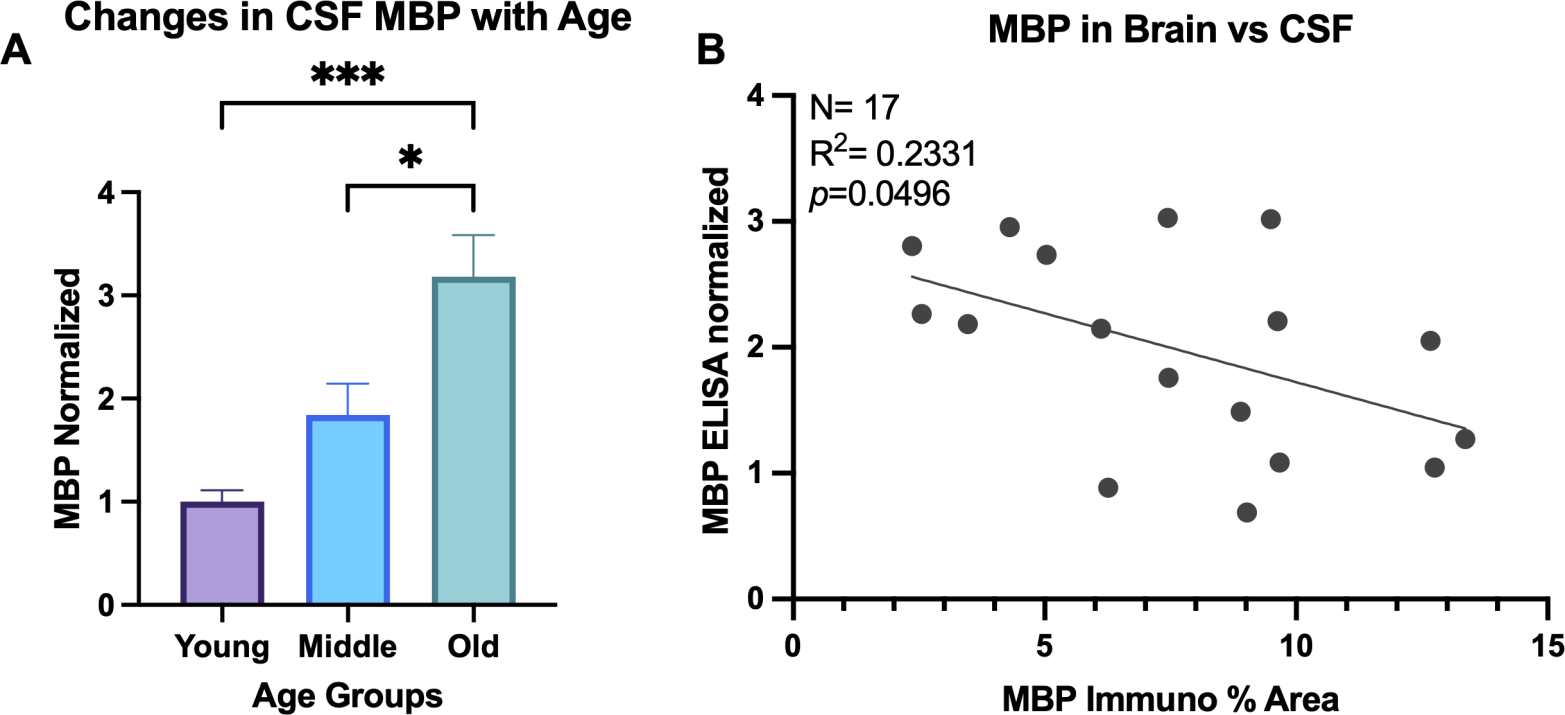
MBP in CSF increases with age. **(A)** ELISA was run for MBP in CSF samples and concentrations normalized to the young age group. One-way ANOVA analysis revealed MBP increases in old animals compared to both young and middle-aged animals. **(B)** Linear regression comparing the % area of MBP in the brain compared to the normalized concentration of MBP in the CSF of the same animals. *p<0.05; *** *p*<0.001.

It has been reported that the accumulation of myelin damage in the brain overburdens microglia cells so they can no longer keep up with the demand of breaking down the cholesterol-rich molecules (53). Thus, myelin debris that is not engulfed by microglia may be cleared into the CSF with age. To test this, an ELISA for MBP was run on CSF samples from 27 animals. One animal was excluded from analysis due to concentration below the manufacturer’s range and 3 animals were excluded after they were identified as outliers on the ROUT outlier test on GraphPad Prism. One-way ANOVA results show significant change in MBP in CSF with age [F(2,20)=10.24, *p*=0.0009]. Specifically, Tukey’s *post hoc* test revealed significant increase of MBP in the CSF of old animals compared to both young (*p*= 0.0008) and middle-aged animals (*p*= 0.0279), as shown in **Figure 6A**. This is consistent with the hypothesis that myelin breakdown and clearance into the CSF increases with age. No change was found comparing young and middle-aged animals (*p*= 0.2847; **Figure 6A**). Linear regression analyzing the % area of MBP in the brain and concentration of MBP in the CSF revealed significant correlation, where less MBP in the brain was associated with more MBP in the CSF [F(1,15)= 4.56, R^2^ = 0.2331, *p*=0.0496] (**Figure 6B**). Together, these suggest that elevated myelin damage in the brain is cleared through the CSF across the lifespan, with highest levels of damage in the old age group.

## Discussion

### Summary of results

While the root cause of myelin pathology that occurs with age remains unknown, a likely candidate is microglia that become reactive and dysregulated with age-related inflammation. Here, we investigated changes in immunologic proteins C1q and CD47 that modulate microglia phagocytosis of cellular material relative to age-related myelin damage. Our main findings demonstrate that in the normal aging monkey brain, the “eat me” signal C1q, is increased both in the cingulum bundle microenvironment and specifically localized to myelin, which it may be tagging for phagocytosis. Of particular interest, the “don’t eat me” protein CD47 that could mitigate C1q effects decreased in middle age, which would effectively increase myelin vulnerability. However, this was followed by a paradoxical increase of CD47 in old age. *In situ* hybridization showed that CD47 RNA expression decreased in oligodendrocytes with age. Further, we found that with age, microglia take on more reactive phenotypes along with increased expression of C1q RNA. Finally, these changes in C1q, CD47, and microglia phenotypes correlate with more severe cognitive impairment. Together, these results suggest that both C1q and CD47 are dysregulated with age and likely contribute to both the increase in microglia reactivity and vulnerability of myelin to phagocytosis that is associated with cognitive impairment.

### Oligodendrocyte and microglia roles in myelin damage

Oligodendrocytes and microglia work together to maintain myelin homeostasis and our observed immune dysregulation makes both likely contributors to age-related myelin deterioration. Mature oligodendrocytes capable of myelinating differentiate from oligodendrocyte precursor cells (OPCs) to maintain myelin across the lifespan (60). Mature oligodendrocytes accumulate damage with age and are particularly vulnerable to oxidative stress byproducts after long periods of time under the extreme metabolic demand required to maintain myelin (60). Thus, myelination by mature aged oligodendrocytes results in the production of abnormal myelin (58). We showed loss of MBP in the cingulum bundle, which not only indicates loss of myelin but also the inability of oligodendrocytes to produce compact myelin with tight lamination as MBP facilitates adhesion of myelin surfaces (49).

Our data show that CD47 RNA expression was decreased in oligodendrocytes with age, suggesting a down-regulation of the “don’t eat me” signal. Furthermore, we have recently reported that aged OPC numbers do not change but OPCs exhibit impaired ability to differentiate into mature myelinating oligodendrocytes, likely resulting in less myelin production and ultimately myelin loss (61). Previous work demonstrates OPCs contribute to remyelination by differentiating into mature, myelinating oligodendrocytes (62). Thus, OPCs that fail to differentiate cannot replace the damaged aged oligodendrocytes. Therefore, defective myelin would continue to be produced, making the cause for this OPC differentiation failure an important question to be addressed.

Microglia directly signal to OPCs to promote proliferation, differentiation, and subsequent remyelination (63). During demyelination, regenerative or “M2” microglia drive oligodendrocyte differentiation to facilitate efficient remyelination (64). Additionally, efficient microglia clearance of myelin debris is critical for remyelination (23). However, we demonstrated that microglia reactivity increases with age, suggesting microglia may not be able to polarize into a regenerative phenotype that facilitates OPC differentiation required for remyelination. Further, with age, microglia become overburdened with myelin debris (53) resulting in the accumulation of myelin debris which has been shown to obstruct OPC proliferation (65). Moreover, aged microglia upregulate C1q, which has been shown to directly inhibit OPC differentiation into mature oligodendrocytes via the Wnt signaling pathway in a cuprizone model of MS and myelin pathology (66). Here, we found increased C1q in aging white matter, which may also be inhibiting OPC differentiation. Thus, rather than promoting remyelination, microglia contribute to the inhibition of remyelination during aging by increasing C1q levels, which could lead to increased phagocytosis of myelin and obstruct OPC differentiation as well as ineffectively clearing myelin debris.

### C1q and CD47 signaling in disease states

Dysregulation of C1q and CD47 may play an active role in microglia phagocytosis dysregulation and worsening of subsequent pathology during aging. C1q is a pattern recognition receptor (PRR) that directly binds to structures releasing disease associated molecular patterns (DAMPs) associated with cellular damage or debris (57). C1q binding initiates the classical complement cascade of downstream molecule recruitment that ultimately activates innate immune cell response to remove the selected structure (67, 68). Chronic upregulation of C1q occurs in age-related diseases such as AD and MS in association with excess microglia phagocytosis (69). Excessive C1q-stimulated phagocytosis exacerbates disease pathophysiology such as synapse loss in AD and demyelination in MS that contributes to cognitive and neurologic impairment (70–72). In mice, microglia mediate forgetting in a complement dependent manner (73), and elevated C1q in normal aging monkeys and rodents is associated with cognitive impairment (34, 36, 37, 74). The present results confirm elevated C1q with age in white matter tracts where it may contribute to the pro-inflammatory environment and myelin damage. Further, we demonstrate here, along with our previous study in gray matter (37), the association of both microglia reactivity and elevated C1q levels with poorer cognitive performance. Our results show that there is a significant increase in C1q localized to myelin during aging, suggesting that more myelin is targeted by this “eat me” signal which could exacerbate myelin pathology and associated age-related cognitive impairment. Taken together, this suggests aberrant C1q expression and microglia reactivity may underly age-related cognitive decline in learning and memory.

The inhibitory signal, CD47, is a neuroimmune regulatory protein (NIReg) involved in suppressing the duration of inflammation and promoting tissue recovery (32, 33). CD47 binds with receptor signal regulatory protein (SIRPα) located on microglia cells to reduce unnecessary phagocytosis of structures such as myelin (35, 39, 75). Loss of proper CD47-SIRPα interactions has been shown to contribute to progression of neurodegenerative disease including stroke, AD, and MS (32). CD47 reduction is associated with active MS lesions and toxic amyloid beta activity in AD (76–78). The results reported here suggest that CD47 may be similarly involved in the myelin damage of normal aging. We report that levels of CD47 did not change in the cingulum bundle with age, but that colocalization with myelin decreases in middle age at onset of myelin damage but paradoxically increases again with age when our earlier studies have shown that myelin damage increases (18, 19, 79). From our results, it is unclear how CD47 levels remain stable in the cingulum bundle with age while there is decreased expression of CD47 mRNA in oligodendrocytes. Interestingly, recent work has demonstrated that neuronal SIRPα plays a role in regulating microglia phagocytosis by modulating microglial interaction with CD47 during synaptic refinement and in neurodegeneration (80, 81). Thus, it is possible that the dynamic between CD47 and neuronal SIRPα is altered with aging and should be investigated in future studies. Further, the role of CD47 in disease pathogenesis is less understood and appears to be conflicting. For instance, CD47 may become detrimental in disease states where swift debris clearance and inflammatory response are needed, but the absence of CD47 promotes inflammation and phagocytosis (32). In an EAE model of CD47, blocking of CD47 at disease initiation attenuated disease progression while blocking CD47 at peak demyelination worsened disease states (76). The same is true regarding myelin where deletion of CD47 results in quicker and faster myelin clearance following acute peripheral nerve damage (82), but CD47 knockout accelerates myelin disruption and dismantling by myelin producing Schwann cells (82). As in disease states, our results suggest that CD47 similarly plays nuanced roles in myelin degeneration.

### Microglia-mediated myelin phagocytosis in aging

Normal myelin turnover involves the balance between myelin degradation and remyelination which is crucial for myelin homeostasis in the adult brain (83, 84). Disruption of this process, whether by excess myelin phagocytosis or inefficient myelin clearance has severe consequences including triggering inflammation, accumulation of toxic debris, blockage of remyelination, and ultimately worse myelin damage (83), as seen during aging. While aged microglia are still able to phagocytose myelin, their engulfing capacity decreases (85). Further, microglia exhibit difficulty degrading lipid-rich myelin, and these fragments form insoluble inclusions resembling lipofuscin within microglial lysosomal compartments, impeding efficient breakdown (53). Our data show that microglia become more reactive with age as more microglia exhibit a hypertrophic morphology and express C1q and Gal-3. As reported by Safaiyan et al. (86), distinct microglia phenotypes emerge specific to white matter tracts during normal aging reflecting overburdening and chronic degradation of myelin. These microglia, known as white matter associated microglia (WAM), exhibit downregulation of homeostatic genes and upregulation of genes related to lipid metabolism, lysosomal activity, phagocytosis, and major histocompatibility complex class II (MHC-II) (86). Gal-3 is a prominent feature of the WAM signature (86) and may be neurotoxic and involved in myelin debris clearance (86 &87). Indeed, we report the increased proportion of Gal-3 hypertrophic microglia was associated with increased cognitive impairment, which corroborates previous work from our lab (87). Studies of MS in humans demonstrate similar shifts in microglia phenotype from homeostatic to reactive genes. For example, microglia gene expression during active demyelinating lesions for phagocytosis, oxidative injury, and antigen presentation is upregulated (88).

Overall, our data show the heterogeneity of microglial populations that mediate complex interactions within phagocytic signaling (89), which likely have distinct roles in age-related myelin damage clearance. We hypothesized that if myelin debris is accumulating beyond the capacity with which microglia can phagocytose it, excess debris would be cleared into the CSF with age, which is supported by our results. It is important to note that microglia do not act alone in myelin phagocytosis, as astrocytes show limited capability to phagocytose myelin debris, although this may be a compensatory mechanism when microglia become dysfunctional (83, 90). Nevertheless, our results show that microglia become phagocytic and inflammatory with age as myelin damage increases and the “eat me/don’t eat me” signaling becomes dysregulated.

### Limitations and pitfalls

Unlike rodent models, monkey frontal white matter tracts have dense white matter, making reliable quantification of myelin difficult using myelin staining, and electron microscopy was not available for the cases used here so quantification of myelin damage was not possible. However, immunolabeling of MBP in the cingulum bundle enabled the quantification of % area of signal with age as surrogate for degree of myelin damage. Furthermore, we measured MBP in the CSF to assess the amount of myelin cleared into the CSF as a potential marker for myelin damage and found that it increased with age. The use of the rhesus monkey is a highly valuable model as these primates have cytoarchitecture highly similar to the human brain, including dense white matter tracts, and similar age-related cognitive impairment despite being spared from AD. Thus, we can extrapolate cellular changes during age-related cognitive decline without the confound of neuron loss in AD. Despite the necessarily correlative nature of the data presented here, the association of cellular changes affecting myelin and cognitive impairment provides valuable insights into potential therapeutic targets.

## Conclusions

The observations presented here show that both C1q and CD47 are dysregulated during normal aging in the primate brain in association with myelin breakdown in white matter and cognitive decline. Here, we showed an increase in C1q in the cingulum bundle and specifically on myelin, where it potentially acts as an “eat me” signal to microglia to facilitate myelin phagocytosis. Concurrently, microglia exhibited a higher frequency of reactive phenotypes with age, providing evidence of microglia phagocytic activity coinciding with complement colocalization to myelin. Additionally, the mitigation of C1q effects by the “don’t eat me” molecule CD47 is reduced in aging as CD47 colocalization to myelin decreased in middle age and oligodendrocyte CD47 mRNA expression decreased with old age. Together, this leaves myelin and oligodendrocytes vulnerable to tagging by C1q and phagocytosis by microglia. We believe the changes in C1q and CD47 reported here are detrimental to white matter during aging as they are correlated with worsening cognitive impairment. Our findings lay the foundation for future work investigating the mechanisms by which both “eat me” and “don’t eat me” signals contribute to age-related myelin damage, microglia reactivity, and cognitive decline. While C1q and CD47 are just two molecules in a large cascade of signals, they should be considered as potential targets for therapeutic interventions aimed at slowing cognitive aging.

## Data Availability

The raw data supporting the conclusions of this article will be made available by the authors, without undue reservation.
